# The Emerging Role of Extracellular Vesicles in Endocrine Resistant Breast Cancer

**DOI:** 10.3390/cancers13051160

**Published:** 2021-03-08

**Authors:** Giusi La Camera, Luca Gelsomino, Amanda Caruso, Salvatore Panza, Ines Barone, Daniela Bonofiglio, Sebastiano Andò, Cinzia Giordano, Stefania Catalano

**Affiliations:** 1 Department of Pharmacy, Health and Nutritional Sciences, University of Calabria, Via P. Bucci, 87036 Arcavacata di Rende, CS, Italy; lcmgsi93e44d086t@studenti.unical.it (G.L.C.); luca.gelsomino@unical.it (L.G.); crsmnd96t42d086q@studenti.unical.it (A.C.); salvatore.panza@unical.it (S.P.); ines.barone@unical.it (I.B.); daniela.bonofiglio@unical.it (D.B.); sebastiano.ando@unical.it (S.A.); 2 Centro Sanitario, University of Calabria, Via P. Bucci, 87036 Arcavacata di Rende, CS, Italy

**Keywords:** breast cancer, endocrine resistance, extracellular vesicles, exosomes, targeted therapies

## Abstract

**Simple Summary:**

Two-thirds of breast cancer patients present an estrogen receptor–positive tumor at diagnosis, and the main treatment options for these patients are endocrine therapies such as aromatase inhibitors, selective modulators of estrogen receptor activity or selective estrogen receptor down-regulators. Although endocrine therapies have high efficacy in early-stage breast cancers, the failure of the therapeutic response to these hormonal treatments remains the major clinical challenge. Recently, extracellular vesicles (EVs) have emerged as a novel mechanism of drug resistance. Indeed, EVs isolated from tumor and stromal cells act as key messengers in intercellular communications able to propagate traits of resistance and/or educate the microenvironment to sustain a breast cancer resistant phenotype. Understanding the EV-mediated molecular mechanisms involved in hormonal resistance can provide the rationale for novel and effective treatment modalities and allow for the identification of potential biomarkers to monitor therapy response in ER-positive breast cancer patients.

**Abstract:**

Breast cancer is the most common solid malignancy diagnosed in females worldwide, and approximately 70% of these tumors express estrogen receptor α (ERα), the main biomarker of endocrine therapy. Unfortunately, despite the use of long-term anti-hormone adjuvant treatment, which has significantly reduced patient mortality, resistance to the endocrine treatments often develops, leading to disease recurrence and limiting clinical benefits. Emerging evidence indicates that extracellular vesicles (EVs), nanosized particles that are released by all cell types and responsible for local and systemic intercellular communications, might represent a newly identified mechanism underlying endocrine resistance. Unraveling the role of EVs, released by transformed cells during the tumor evolution under endocrine therapy, is still an open question in the cancer research area and the molecular mechanisms involved should be better defined to discover alternative therapeutic approaches to overcome resistance. In this review, we will provide an overview of recent findings on the involvement of EVs in sustaining hormonal resistance in breast cancer and discuss opportunities for their potential use as biomarkers to monitor the therapeutic response and disease progression.

## 1. Introduction

Breast cancers are heterogeneous and dynamic diseases classified into molecularly distinct subtypes based on the expression of estrogen receptor (ER), progesterone receptor (PR) and human epidermal growth factor receptor 2 (HER2), which influence the clinical outcomes and the therapeutic approaches [1]. Approximately two-thirds of all breast cancers express the ER (ER-positive) and depend on its functionality for their proliferation and survival. ER is a member of the nuclear hormone receptor superfamily that, as a ligand-activated transcription factor, regulates the expression of target genes by binding with specific estrogen response elements (EREs), termed “classical” genomic activity [2]. However, estrogen binding to the receptor can also activate different molecular mechanisms termed “nonclassical” ER-mediated transcriptional regulation [3,4,5]. This occurs via ERE-independent signaling in which ER interacts with other transcription factors, such as activator protein-1 (AP-1), specificity protein 1 (Sp1) and nuclear factor-κB (NF-κB), thus modulating downstream gene expression [3,4]. Moreover, ER activity can be mediated in a ligand-independent manner by a functional cross-talk with growth factor (GF) signaling pathways able to induce post-transcriptional ER modifications (e.g., phosphorylation, acetylation, methylation) that in turn regulate receptor activity [6,7,8]. Considering the crucial role of the estrogen/ER axis in breast cancer biology, the endocrine-based therapies are currently the primary treatment used in ER-positive breast cancer patients. The therapies include inhibitors of the aromatase enzyme (AIs, e.g., anastrozole, letrozole and exemestane), selective ER modulators (SERMs, e.g., tamoxifen and raloxifene) or selective ER downregulators (SERDs, e.g., fulvestrant) [9,10]. Endocrine therapies have high efficacy in early-stage breast cancers, and clinical benefit is achieved in about 50% of metastatic tumors [11,12]. However, despite the high sensitivity of ER-positive breast cancer subtypes to endocrine therapies, a large number of patients result resistant to these therapeutic interventions, experiencing disease recurrence either during or after completion of treatments [10]. Although several mechanisms have been proposed to contribute to the emergence of resistant phenotypes, the complete characterization of the drivers of endocrine resistance in breast cancer is still an open question to explore [13].

Recently, it has become apparent that secreted extracellular vesicles (EVs), as key determinants of cell-to-cell communication, play a pleiotropic role in a wide variety of physiological and pathological processes, including carcinogenesis. EVs are nanosized particles, enclosed within a phospholipid bilayer membrane, that based on their size and cellular origin can be divided into subgroups of “small” vesicles, called exosomes, vesicles that are slightly larger (microvesicles/ectosomes) and apoptotic bodies [14,15]. EVs, released both by normal and neoplastic cells are able to modulate the phenotypic behavior of recipient cells by transferring their genetic and molecular cargo. Current research reported that EVs regulate the complex intercellular pathways involved in breast cancer tumorigenesis from tumor initiation and progression towards metastatic disease and drug resistance [16]. Indeed, data from pre-clinical and clinical specimens’ studies indicate that EVs secreted from both tumor and stromal cells through their functional cargo (proteins, mRNAs, miRNAs, DNAs) contribute to breast cancer drug resistance, regulating several processes, including cell survival, epithelial–mesenchymal transition, a stem-like phenotype, education of the tumor microenvironment and drug metabolism [17,18]. Therefore, understanding the EV-mediated molecular mechanisms and signaling pathways that promote therapy resistance and identifying potential biomarkers to predict and monitor the therapeutic response are necessary for a more effective breast cancer treatment.

Here, we will review the emerging data in understanding the role of EVs in the mechanisms of resistance to endocrine manipulation and to the alternative strategies implemented to overcome endocrine resistance in ER-positive breast cancer. Given the lack of standardized nomenclature and isolation protocols for a large family of vesicles, we will use the term EVs to refer to this heterogeneous population throughout this review.

## 2. Overview of Extracellular Vesicles

Extracellular vesicles (EVs) are multi-signal messengers naturally released by all cell types into the extracellular space that can be recovered from common biological fluids, such as blood, urine, breast milk, seminal fluids, saliva and malignant effusion [19,20,21,22,23,24]. Initially observed in plasma by Chargaff and West in 1946 as platelet-derived particles and then described by Wolf in 1967 as “platelet dust”, the EVs are now well recognized as key players in intercellular communication in both physiological and pathological conditions. The generic name “extracellular vesicles” defines a large population of lipid bilayer-enclosed extracellular structures that can be classified on their physical and biochemical characteristics, cellular source and biogenesis pathways. Based on the “Minimal Information for Studies of Extracellular Vesicles” (MISEV) guidelines [15,25], the EV population can be divided into subgroups of “small” vesicles/exosomes, microvesicles/ectosomes and apoptotic bodies. Particularly, microvesicles (MVs) and exosomes are the two major subtypes of EVs that have received considerable attention in recent years for their ability to induce phenotypic reprogramming in recipient cells [26]. MVs or ectosomes represent the larger EV population (100–1000 nm in diameter) that bud directly from the plasma membrane. Exosomes, the smallest subtype of EVs (30–150 nm in diameter) generated within endocytic compartments, are secreted into the extracellular space after the fusion of multivesicular bodies (MVBs) with the plasma membrane. Exosome biogenesis can be driven by the activity of the Endosomal Sorting Complex Required for Transport (ESCRT) machinery [27,28], and many pieces of evidence also suggest the existence of an ESCRT-independent pathway that involves tetraspanins, lipids and RabGTPases [29]. The mechanism of EV biogenesis can be modulated by different extracellular conditions, such as hypoxia, the Ca2+-dependent pathway, growth factors and adipokines [30,31,32,33]. EV cargo is composed of a common subset of proteins involved in membrane transport and fusion processes, such as Rab GTPases, annexin, tetraspanins (CD9, CD63, CD81), integrins, adhesion molecules, heat shock proteins, enzymes, matrix metalloproteinases, glycoprotein receptors and immune regulator molecules (MCH-I and -II), but also includes selected proteins, lipids and nucleic acids that reveal the unique molecular signature of the cells of origin [29,34].

The release of EVs is increased in cancer [35,36]. Particularly, it has been reported in breast cancer that the secretion of EVs was significantly higher in transformed cells than in normal mammary epithelial cells [37]. Similarly, analysis of particle concentration revealed an increase in EV number in the plasma of patients with stage I-IV breast cancer compared to healthy control subjects, while no significant differences were found between the EV number of “in situ” breast cancer and the control group [38]. More recently, Stevic et al. reported an increased amount of vesicles in blood samples of women with Triple Negative Breast Cancer compared to the number of vesicles in plasma samples from healthy women [39]. Overall, these findings suggest the possibility that more aggressive breast cancers could release a large amount of vesicles, but the comparison between the amounts of circulating EVs at different stages of disease should be better defined and deserves more investigations. Breast tumor-derived EVs have been reported to have a role in all cancer hallmarks controlling a wide range of pathways and regulating gene expression by transferring their intra-vesicular content (proteins, lipids, enzymes, metabolites, DNA, mRNA, miRNA, long and short non-coding RNA) into recipient cells [40]. In addition to regulating invasion, vessel formation, pre-metastatic niche preparation, and immune surveillance escape, the bioactive molecules carried by EVs have been proposed as important players in the mechanisms of resistance to therapeutic treatments [41,42] and might represent promising candidate biomarkers for breast cancers [43,44].

## 3. EVs and Therapeutic Resistance in ER-Positive Breast Cancer

**Figure 1:**
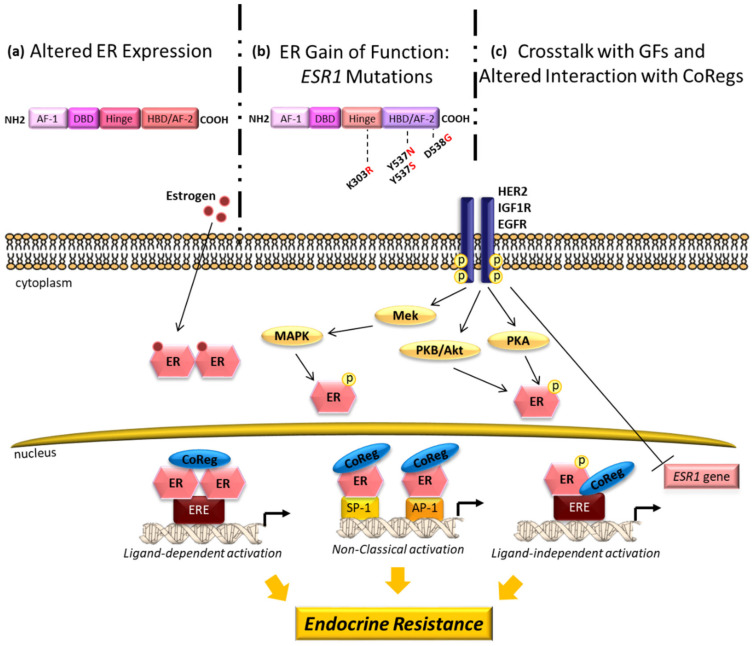
Representation of the main mechanisms sustaining endocrine resistance mediated by estrogen receptor α (ERα). The structural domains of ERα contain the ligand-independent activation function (AF-1) in the amino-terminal region, a DNA-binding domain (DBD), and a carboxy-terminal hormone-binding domain (HBD), containing the ligand-dependent activation function (AF-2). In the classical ERα activation, estradiol binds to its cognate receptor in the cytoplasm, leading to dimerization, nuclear translocation and interaction with specific DNA sequences (ERE, estrogen responsive element) in target genes (ligand-dependent activation). The ERα can also bind to transcription factors such as activation protein 1 (Ap1) and specificity protein 1 (Sp1) activating gene target transcription (non-classical activation). ERα signaling activation can also occur through second messengers downstream of growth factor signaling pathways (ligand-independent activation). (**a**) Altered ERα expression including either loss of ERα or increased ERα expression. (**b**) Gain of function mutations in the ESR1 gene. The most characterized mutations within ERα were reported. The K303R somatic mutation, in the hinge domain, allows ERα to be more highly phosphorylated by Protein Kinase A (PKA) and Protein Kinase B (PKB/Akt), while the Y537N, Y537S, and D538G mutations, in the HBD/AF-2 domain, allow the receptor to be phosphorylated by Mitogen-activated protein kinase (MAPK), resulting in a ligand-independent constitutive activation of the receptor. (**c**) An increased bidirectional cross-talk between wild-type or mutated ERα and growth factor receptors (epidermal growth factor receptor-EGFR, the human epidermal growth factor receptor 2-HER2, the insulin-like growth factor receptor 1-IGFR 1) induces several downstream phosphorylation events that affect ERα activation, and an altered interaction of ER with coregulators affects ER transcriptional activity in a ligand-independent manner sustaining endocrine resistance. Growth factor signaling can also contribute to endocrine resistance diminishing *ESR1* gene expression.

Endocrine-targeted treatments represent the mainstay of the standard care both in the adjuvant and recurrent settings of ER-positive breast cancer patients. However, the reduction in the effectiveness of endocrine regimens is one of the major obstacles to successful treatment and still represents an essential clinical challenge for the management of ER-positive disease. Commonly, resistance has been classified into primary (or “de novo” resistance), where insensitivity already exists before treatment, and secondary (or acquired resistance) that develops in patients initially responding to endocrine therapy. The mechanisms of endocrine resistance in breast cancer are very complex and a plethora of molecules and escape signaling pathways have been involved [45,46]. Endocrine insensitivity due to the loss of ER expression has been reported in less than 20% of metastatic breast cancers [47,48], while the majority of endocrine-resistant tumors still retain the ER expression and activity during the development of resistance [49,50]. Indeed, ER signaling primarily mediated by ligand-independent receptor activation [51], remains crucial in mediating resistance. Several mechanisms are responsible of sustaining ER activity in resistance including: (i) an increased expression of the receptor itself [52]; (ii) the gain of function mutations in *ESR1* gene [53,54,55,56,57,58]; (iii) an altered interaction of the receptor with coregulators (coactivators and/or corepressors) [59,60,61]; (iiii) an increased bidirectional cross-talk between ER and growth factor receptor/oncogenic kinase signaling pathways [62]. In addition, growth factor signaling can contribute to the transcriptional repression of ER gene expression resulting in endocrine resistance [63,64,65]. The main described mechanisms involved in these molecular events are summarized in Figure 1.

Furthermore, resistance to endocrine therapies could be associated with the development of cellular characteristics similar to those of cells undergoing epithelial-to-mesenchymal transition (EMT) [66], with cells expressing a cancer stem-like phenotype [67,68], or remaining dormant in a quiescence state for a long time in the body before re-awakening [69]. Indeed, it has been demonstrated that dormant cells express features that support their survival despite anti-proliferative endocrine therapy [70]. Moreover, a growing amount of evidence supports the concept that extrinsic resistance might arise from the interplay between tumor cells and several components of the tumor microenvironment, including cancer-associated fibroblasts (CAFs), inflammatory and immune cells, extracellular matrix (ECM), soluble factors and EVs [71,72,73]. Despite the advances in the knowledge of resistance mechanisms, recently the clinic-genomic characterization of endocrine-resistant advanced breast cancer revealed the lack of a known mechanism of resistance to hormonal therapy in 60% of analyzed tumors [13]. Nowadays, it has become increasingly clear that EV-mediated cell communication represents a new identified mechanism underlying endocrine resistance. The key findings outlined by the current literature in this field are summarized in Table 1.

### 3.1. EVs and Hormonal Resistance

Semina et al. initially found that co-culturing estrogen-dependent MCF-7 breast cancer cells with the MCF-7 cell line resistant to tamoxifen (MCF7/T) induced horizontal hormone resistance in hormone-sensitive cells as a result of intercellular interaction, suggesting the possible involvement of EVs in the progression of hormonal resistance [74]. More recently, the same authors demonstrated that long-term treatment of MCF-7 breast cancer cells with EVs from MCF7/T caused the partial resistance of sensitive cells to this antiestrogen. These effects were associated with a decreased ERα activity along with an activation of transcriptional factors involved in growth, apoptosis and EMT processes, such as AP-1, NF-κB and SNAIL1 in both the primary resistant cells and the cells with the EV-induced resistance. Besides, a marked increase in the expression and activity of Akt in all of the resistant cells compared to the parental MCF-7 cells was shown, and exposure to a PI3K inhibitor prevented the EV-induced resistance, highlighting the involvement of PI3K/Akt signaling in the EV-transferring resistance [75]. Recently, it has been shown that in the MCF-7-LTED (Long-Term Estrogen-Deprived) subline, modelling resistance to Aromatase Inhibitors (AIs) is associated with an enhanced EV production, which appears to be related to an increased Rab GTPase expression. Quantitative proteomic analysis showed an enrichment of proteins frequently identified in vesicles in MCF-7-LTED compared to MCF-7 cells. Interestingly, the most up-regulated proteins in MCF-7-LTED cells belong to the Rab GTPase family, important regulators of vesicle biogenesis and secretion in cancer [76].

EVs, released by both tumor and stromal cells, can confer resistance to therapy-sensitive cancer cells by transmitting miRNAs, and small non-coding post-transcriptional regulators of gene expression miRNAs can accumulate in EVs, where they are protected from cleavage by RNAses in the blood [89]. EVs released from tamoxifen-resistant MCF-7 cells by transferring miR-221/222 have been reported as a mechanism of tamoxifen resistance. It has been demonstrated that the crucial vesicle component miR-221/222 can effectively reduce expression of their target genes P27 and ERα, leading to enhanced tamoxifen resistance [77]. More recently, Sansone et al. demonstrated that CAF-derived EVs, by transferring miR-221 to breast cancer cells, lead to an expansion of the cancer stem cell-like population promoting hormone therapy resistance (HTR). Indeed, they found in patients with HTR metastatic disease a higher expression of miR-221 in circulating EVs compared with healthy controls. Mechanistically, they reported that autocrine IL6/Stat3 signaling contributes to the proliferation of CAFs and to the biogenesis of oncomiR-221^hi^ EVs. Interestingly, the depletion of murine CAFs in patient-derived xenografts from breast cancer bone metastases restored sensitivity to HT associated with a reduction in the cancer stem cell-like phenotype. On the contrary, in HT-sensitive cancer cells, both murine and human CAFs induced “de novo” HT resistance through the induction of the Notch-mediated breast cancer stem cell-like phenotype that expressed low levels of ER [78]. The EV-mediated transfer of long non-coding RNA (lncRNA) has been demonstrated as an additional mechanism of tamoxifen resistance. Xu et al. found increased levels of lncRNA urothelial cancer-associated 1 (UCA1) in the tamoxifen-resistant variant of MCF-7, termed LCC2, and also in EVs released from these cells. MCF-7 cells pretreated with EVs/LCC2 exhibited, after tamoxifen treatment, an enhanced cell viability, a reduced expression of cleaved caspase-3 and a lower ratio of apoptosis. Knockdown of UCA1 in LCC2 cells clearly indicated that this lncRNA plays a crucial role in inducing tamoxifen-resistance. Indeed, vesicles from UCA1-knockdown LCC2 cells showed a significantly reduced capability to promote tamoxifen resistance in MCF-7 cells [79].

Several findings have shown that EVs are able to modulate the key steps of the metastatic process [14]. A recent work reported a link between EVs and the P2X purinoreceptor 7 (P2X7) proposing a new mechanism of metastasis in tamoxifen-resistant (TAMR) breast cancer cells. P2X7, a ligand-gated ion channel receptor activated by ATP, is over-expressed in several tumors, including breast cancer, where it is involved in tumor development and metastasis [90,91,92]. The authors reported that EVs isolated from TAMR-MCF-7 cells increased their own migratory capabilities in a concentration-dependent manner. Moreover, P2X7 antagonist decreased the number of secreted EVs and the protein levels of CD63 in TAMR-MCF-7 cells, highlighting the crucial role of P2X7 in influencing the EV production [80]. Sansone et al. have provided data supporting the hypothesis that the horizontal transfer of mitochondrial DNA (mtDNA) from EVs regulates escape from dormancy in hormonal therapy (HT)-resistant breast cancer leading to metastatic progression. They found the full mitochondrial genome packaged in CAF-derived EVs and in circulating EVs from patients with HT-resistant metastatic disease. Specifically, CAF-derived EVs induced an escape from metabolic quiescence in both HT sensitive cells or HT metabolically dormant populations. The horizontal transfer of mtDNA was observed in cancer stem-like cells and associated with an increased self-renewal potential leading to endocrine therapy resistance [81].

### 3.2. EVs and Therapeutic Strategies to Overcome Endocrine Resistance

Mechanisms of endocrine resistance involve extensive cross-talk between ER and tyrosine kinase growth factor receptors and their downstream signaling pathways. Genetic or epigenetic alterations in various components of the signaling pathways, such as overexpression of human epidermal growth factor receptor 2 (HER2) and aberrant expression of cell-cycle regulators, have been reported. Indeed, it is well established that ongoing endocrine treatment can induce adaptive changes in breast cancer cells resulting in an aberrant activation of growth factor-mediated proliferative and survival pathways such as PI3K (Phosphoinositide 3-Kinase) Akt/mTOR (mechanistic target of rapamycin) and Rat sarcoma viral oncogene (RAS)/Mitogen-activated kinase kinase (MEK)/MAPK which in turn are able to induce an estrogen-independent receptor activation [93]. The benefit of combining endocrine therapy with molecular targeted agents and signal transduction inhibitors has been the major focus of clinical trials to overcome or delay endocrine resistance in ER-positive breast cancer [45].

#### 3.2.1. EVs and HER2 Targeted Therapy

Multiple clinical and experimental observations have largely associated the high expression and activation levels of epidermal growth factor receptor family signaling pathways, especially of the HER2, with lower therapeutic efficacy of endocrine therapy [94,95,96,97]. The membrane tyrosine kinase *ERBB2* gene has been found amplified in ~25% of ER-positive breast cancer, and clinical evidence revealed that these HER2-positive tumors display the more aggressive behavior of the disease, resistance to hormonal therapy and shorter overall survival [98,99,100,101]. These well-established contributions of epidermal growth factor receptor signaling pathways in the development of resistance to endocrine therapies represent the rationale behind combined treatments with endocrine therapy and selective target inhibitors of growth factor signaling pathways [10,102]. Although treatment with the anti-HER2 monoclonal antibody trastuzumab results in a good clinical response in breast cancer patients, not all HER2-overexpressing tumors respond to these therapies and many of these that initially respond later acquire resistance.

Some studies reported the involvement of EVs in promoting resistance to targeted therapy. An initial study demonstrated the possibility that EVs affect sensitivity to HER2-targeted therapy “in vitro” and “in vivo”. Particularly, it has been found that EVs isolated either from the ER-positive BT474 cell line overexpressing HER2 or in breast cancer patients’ serum can directly bind to the anti-HER2 monoclonal antibody trastuzumab and reduce its bioavailability [82]. Another study showed that HER2-targeted drug resistance is correlated with an increased amount of transforming growth factor beta 1 (TGF-β1) and programmed death-ligand 1 (PD-L1) and resistance to the anti-tumor immune response. EVs carrying these molecules can transfer phenotypic traits of cells of origin to drug-sensitive cells, further promoting immune evasion. Moreover, in HER2-positive breast cancer patients, EV-associated TGF-β1 levels correlate with response to HER2-targeted therapy, proposing TGF-β1 as an EV-associated biomarker to monitor the treatment response [83].

EV transmitted non-coding RNAs may also contribute to the trastuzumab resistance. It has been demonstrated that lncRNA small nucleolar RNA host gene 14 (SNHG14) was up-regulated in trastuzumab-resistant ER-positive breast cancer cells. Extracellular lncRNA-SNHG14 was able to be incorporated into vesicles, transferred to sensitive cells and induce trastuzumab resistance by targeting Bcl-2/Bax signaling and thus inhibiting cell apoptosis. Furthermore, the expression level of SNHG14 in circulating EVs was increased in patients who exhibited resistance to trastuzumab, compared with those exhibiting a response, suggesting lncRNA-SNHG14 in serum vesicles as a potential diagnostic biomarker for breast cancer [84]. It has also been shown that an enhanced expression of lncRNA AGAP2 antisense RNA 1 (AGAP2-AS1) in transtuzumab-resistant ER-positive breast cancer cells promotes resistance of recipient cancer cells through packaging into vesicles [85]. Similarly, it has been evidenced that IncRNA actin filament-associated protein 1 antisense RNA 1 (AFAP1-AS1) induces trastuzumab resistance through binding with AU-binding factor 1 (AUF1) protein which enhanced the translation of the *ERBB2* gene [86]. Moreover, Han et al. utilizing publicly available miRNA expression profiling data of breast cancer, identified miR-567 among the dysregulated miRNAs in trastuzumab-resistant cells. They found that an increased miR-567 expression inhibited autophagy by targeting a key autophagy-related protein (ATG5), thus reversing trastuzumab resistance of breast cancer, while the knockdown of miR-567 expression induced resistance to anti-HER2 targeted therapy. In addition, extracellular miR-567 is also able to reverse the trastuzumab resistance of recipient cells [87].

#### 3.2.2. EVs and CDK4/6 Inhibitors

**Figure 2:**
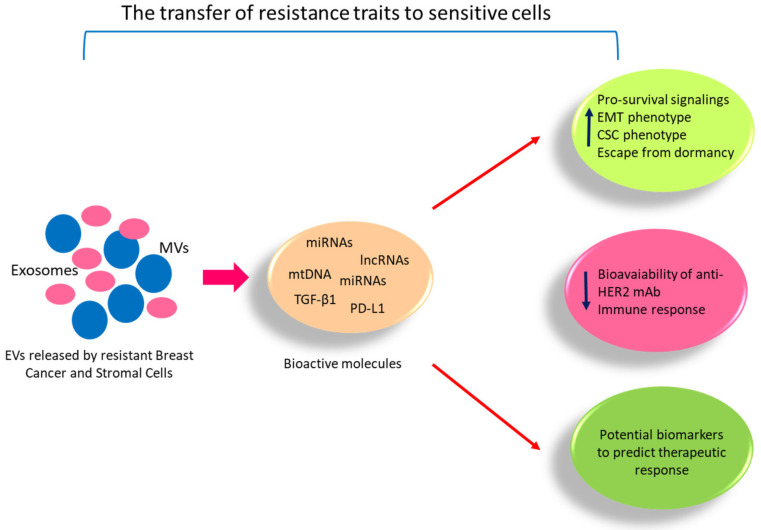
The proposed function of EVs secreted by breast cancer or stromal cells in endocrine treatment resistance. EVs, by transferring their cargo in sensitive breast cancer cells, can confer traits of hormonal resistance by inducing signaling pathways involved in survival, migration, invasion, epithelial mesenchymal transition (EMT), in sustaining the cancer stem cell-like (CSC) phenotype and escape from dormancy. EVs can reduce the bioavailability of anti-HER2 mAb and immune evasion promoting resistance to targeted therapy to overcome endocrine therapies’ failure. Finally, evaluation of the molecular cargo of circulating EVs has promising value to discover potential biomarkers to predict the therapeutic response in ER-positive breast cancer patients.

Since the aberrant expression of cell-cycle regulators that contribute to the loss of cell cycle control [103,104,105,106] has been reported as a mechanism of endocrine resistance, the highly selective cyclin-dependent kinases 4/6 (CDK4/6) inhibitors (e.g., palbociclib, ribociclib and abemaciclib) have emerged as powerful agents to overcome endocrine resistance and have been approved in the metastatic setting for ER-positive/HER2-disease [107]. Despite their clinical benefits, the useful biomarkers able to predict the response to these agents are still lacking. Del Re et al. reported that mRNA expression of thymidine kinase 1 (TK1), CDK4, 6 and 9 in plasma-derived vesicles of ER+/HER2-advanced breast cancer patients can predict sensitivity to CDK inhibitor treatments. Particularly, they found that high CDK4 mRNA levels in EVs are associated with the response to combined treatment of palbociclib plus fulvestrant, while high mRNA levels of TK1 and CDK9 in plasma-derived EVs are associated with resistance to palbociclib treatment [108]. Recently, a role for EVs in mediating CDK4/6 therapy resistance has also been reported [88]. It has been demonstrated that increased CDK6 expression is a hallmark of acquired resistance to CDK4/6 inhibitors and that resistance is conferred through extracellular signalling mediated by EVs. At the molecular level, the authors found that miR-432-5p in EVs transferred from resistant cells to neighboring cells increases CDK6 levels by suppressing the transforming growth factor-β pathway via SMAD4 knockdown, allowing cells to overcome G1 arrest [88]. The modalities by which EVs may influence the therapeutic response in ER-positive breast cancers are summarized in Figure 2.

## 4. Conclusions

The occurrence of resistance to endocrine therapy in ER-positive breast cancer patients still represents the major clinical failure. Considerable evidence suggests that EVs generated from tumor cells in response to stress conditions such as therapeutic treatments might communicate pro-survival messages into recipient cells transferring the ability to escape endocrine treatment. Monitoring the changes in the bioactive molecule profiles of the tumor-derived EVs during the adaptation to the treatment has the unique potential to depict in real-time the dynamic plasticity of tumor evolution during the acquisition of a resistant phenotype. Thus, increasing our understanding of EV-mediated hormonal resistance in ER-positive breast cancers and the translation of these findings to the clinical application can provide a novel and effective treatment modality for future cancer management. Moreover, EVs seem to have high prospects as a potential liquid biopsy and hold great promise for early diagnosis and staging of breast cancer. However, although the high potential for the clinical use of EVs as molecular markers of disease has been suggested, some issues for routinely working with them still persist due to their high variability in the biological samples, which makes it difficult to establish a clinical cut-off, and the lack of a highly sensitive analytical platforms able to avoid interference from contaminating factors. Thus, to fully translate EV research into clinically reliable tools for cancer therapeutic applications, the development of highly sensitive single tumor-EV detection methods are still necessary. In addition, future pre-clinical studies are required to prove the “in vivo” transfer of resistance by EVs to breast cancer cells along with clinical studies to validate the putative EV markers associated with the endocrine-resistant phenotype. For instance, to address this latter issue, longitudinal evaluation of circulating EVs in a larger set of pre-and post-therapeutic intervention samples may help to define the useful biomarker to monitor the evolution of sensitivity to endocrine therapies in women with ER-positive breast cancer.

Given the mounting evidence for the role of EVs in many aspects of breast cancer, biology efforts in this field of cancer research hold great promise.

## 5. Review Criteria

Original articles published in the last two decades have been searched in PubMed, using the following search terms: “extracellular vesicles and breast cancer”, “extracellular vesicles and endocrine resistance”, “extracellular vesicles and tamoxifen resistance”, “extracellular vesicles and aromatase inhibitor resistance”, “extracellular vesicles and estrogen receptor down regulators”. A similar search was performed using the term exosome instead of extracellular vesicles. Furthermore, additional relevant original articles related to the endocrine resistance mechanisms in breast cancer have been selected. All the articles selected were English-language full-text papers.

## Figures and Tables

**Table 1:** Mechanisms underlying endocrine resistance in breast cancer mediated by extracellular vesicles (EVs).

Source of EVs	EV Types	Molecules	Type of Resistance	Effects	Ref.
MCF-7 cells	Exosomes	Unknown	Tamoxifen	Decreased ERα activity, increased of Akt, AP-1, NF-kB and SNAIL1 activity	[74,75]
MCF-7-LTED cells	Exosomes	Unknown	Aromatase inhibitors	Increased exosome release from resistant cells	[76]
TAMR-MCF-7 cells	Exosomes	miR-221/222	Tamoxifen	Decreased P27 and ERα expression	[77]
CAFs	MVs	miR-221	Fulvestrant	Increased CSC population	[78]
LCC2 cells	Exosomes	lncRNA UCA1	Tamoxifen	Increased cell viability, reduced apoptosis	[79]
TAMR-MCF-7 cells	sEVs	Unknown	Tamoxifen	Increased cell migration	[80]
CAFs	EVs	mtDNA	Fulvestrant	Promoted escape from metabolic quiescence, increased CSC self-renewal	[81]
BT474 cells Serum of BC patients	Exosomes	Unknown	Trastuzumab	Reduced HER-2 monoclonal antibody bioavaibility	[82]
Serum of HER-2-positive BC patients	EVs	TGF-β1, PD-L1	Trastuzumab	Increased immune evasion	[83]
Serum of BC patients	Exosomes	lncRNA SNHG14	Trastuzumab	Reduced apoptosis	[84]
BT474-TR cells	Exosomes	lncRNA AGAP2-AS1	Trastuzumab	Inhibited trastuzumab-induced cell cytotoxicity	[85]
BT474-TR cells	Exosomes	lncRNA AFAP1-AS1	Trastuzumab	Increased *ERBB2* gene translation	[86]
BT474 cells	Exosomes	miR-567	Trastuzumab	Reversed trastuzumab resistance	[87]
MCF-7 cells T47D cells	Exosomes	miR-432-5p	Palbociclib	Promoted suppression of TGF-β pathway	[88]

EVs, extracellular vesicles; MVs, microvesicles; sEVs, small extracellular vesicles; ERα, estrogen receptor α; AP-1, activation protein-1; NF-kB, nuclear factor-κB; SNAIL1, snail homolog 1; MCF-7-LTED, MCF-7 long-term estrogen-deprived; P27, cyclin-dependent kinase inhibitor P27; TAMR-MCF-7, tamoxifen-resistant MCF-7; CSCs, cancer stem cell-like; CAFs, cancer-associated fibroblasts; LCC2, tamoxifen-resistant subline of the MCF-7 human breast cancer cell; BC, breast cancer; lncRNA UCA1, long non-coding RNA urothelial cancer associated 1; mtDNA, mitochondrial DNA; TGF-β1, transforming growth factor beta 1; PD-L1, programmed death-ligand 1; SNHG14, small nucleolar RNA host gene 14; AGAP2-AS1, AGAP2- antisense RNA1; AFAP1-AS1, actin filament-associated protein 1 antisense RNA1; *ERBB2*, human epidermal growth factor receptor 2; miR, microRNA; BT474-TR, BT47D trastuzumab resistant.
